# The impact of CBz-PAI interlayer in various HTL-based flexible perovskite solar cells: A drift-diffusion numerical study

**DOI:** 10.1016/j.heliyon.2024.e31138

**Published:** 2024-05-11

**Authors:** Selma Rabhi, Talaat A. Hameed, Sasikumar Mayarambakam, M. Khalid Hossain, Karthick Sekar

**Affiliations:** a Laboratory of Semiconductors Material and Metallic Oxides, USTHB, Bab-Ezzouar, 16111, Algiers, Algeria; b Dr. Yahia Fares University of Medea, Medea 26000, Algeria; c Solid-State Physics Department, Physics Research Institute, National Research Centre, 33 El Bohouth St., Dokki, Giza, 12622, Egypt; d Department of Materials Science and Engineering, Johns Hopkins University, Baltimore, MD 21210, USA; e Institute of Electronics, Atomic Energy Research Establishment, Bangladesh Atomic Energy Commission, Dhaka 1349, Bangladesh; f Aix-Marseille Université, CNRS, Institut Matériaux Microélectronique Nanosciences de Provence, Faculté de Saint Jérôme, 13397 Marseille Cedex 20, France

**Keywords:** CBz-PAI interlayer, Flexible PSCs, SCAPS-1D, HTL

## Abstract

In perovskite solar cells (PSCs), the charge carrier recombination obstacles mainly occur at the ETL/perovskite and HTL/perovskite interfaces, which play a decisive role in the solar cell performance. Therefore, this study aims to enhance the flexible PSC (FPSC) efficiency by adding the newly designed CBz-PAI-interlayer (simply CBz-PAI-IL) at the perovskite/HTL interface. In addition, substantial work has been carried out on five different HTLs (Se/Te–Cu_2_O, CuGaO_2_, V_2_O_5,_ and CuSCN, including conventional Spiro-OMeTAD as a reference HTL with and without CBz-PAI-IL), using drift-diffusion simulation to find suitable FPSC design to attain the maximum PCE. Interestingly, PET/ITO/AZO/ZnO NWs/FACsPbBrI_3_/CBz-PAI/Se/Te–Cu_2_O/Au device architecture demonstrates the highest achievable power conversion efficiency (PCE) of 27.9 %. The findings of this study confirmed that the reference device (without IL) displays a large valence band edge (VBE)/highest occupied molecular orbital (HOMO) energy level misalignment compared to the modified interface device (with CBz-PAI-IL that reduces VBE/HOMO level mismatch) that eases the hole transport, simultaneously, it reduces the charge carrier recombinations at the interface, resulting in diminished V_oc_ losses in the device. Furthermore, the influence of perovskite absorber thickness and defect density, parasitic resistances, and working temperature are systematically examined to govern the superior FPSC efficiency and concurrently understand the device physics.

## Highlights

• This work aims to enhance the flexible PSC PCE by adding the CBz-PAI interlayer.
• Spiro-OMeTAD, Se/Te: Cu_2_O, CuGaO_2_, V_2_O_5,_ and CuSCN HTLs were systematically tested.
• PET/ITO/ETL/FACsPbBrI_3_/CBz-PAI/Se/Te: Cu_2_O/Au device shows a higher PCE of 27.9 %.
• The interlayer device reduces VBE/HOMO level mismatch that eases the hole transfer.

## 1 Introduction

The commercialisation of perovskite solar cells (PSCs) has been somewhat delayed due to the instability despite the high yields recorded by PSCs in recent times, where the current record power conversion efficiency (PCE) is 26.1 % [1,2]. Due to the outstanding physical properties of perovskites (i.e., higher absorption coefficient, adjustable bandgap, longer diffusion lengths, lower exciton binding energy, etc.) [[3], [4], [5], [6]], the corresponding PSCs offer high photovoltaic (PV) performance. However, apart from the instability, defects mainly at interfaces (i.e., electron transport layer (simply ETL)/absorber and absorber/hole transport layer (simply HTL)) play a crucial part in PSC efficiency [7]. Moreover, in PSCs, the charge carriers (i.e., e^−^ & h^+^) transportation/collection/extraction depend on the interfaces, significantly influencing the PSC performance [8]. Therefore, interface and/or passivation engineering has recently attracted researchers [7,[9], [10], [11], [12], [13], [14]] to resolve interface issues in PSCs.

Among all 3D perovskite materials, formamidinum lead iodide (FAPbI_3_) received tremendous attention either with and without mixed cation (i.e., Cs)/mixed anion (Br) perovskite composition, such as FACsPbBrI_3_, and this mixed cation halides perovskites brings the modification in the structural, morphological, electrical and optical properties [[15], [16], [17]]. In addition, it shows enhanced stability when exposed to open-air conditions. However, many defects trap states or charge carrier transport blocks are present at the interfaces, specifically at ETL/perovskite absorber and perovskite absorber/HTL interface and even at the grain boundaries of the perovskite absorber, which advances the recombination issues, lessening the PSC PCE. Furthermore, in PSCs, hysteresis behavior and irreversible degradation primarily occur due to the accumulation of e^−^ and h^+^ at the perovskite absorber surface as well as sub-surface close to the interface (ETL/perovskite and perovskite/HTL) [18]. To avoid these issues in PSC, various surface passivating agents, such as salts, have been devoted to efficiently suppressing the non-radiative recombination at the surface/interface [[19], [20], [21], [22], [23], [24], [25]]. Noticeably, voltage loss (V_oc_) occurred in PSCs due to the energy level misalignment between the perovskite absorber/HTL layers, which enhanced recombinations at the interface. Jianfeng Lu et al. studied the effect of benzenethiol derivatives (with –CN, –NO_2_, –SCH_3_, and –OCH_3_) on the perovskite absorber layer to alter the absorber/HTL interface, and their outcomes show that the modification offers an appropriate band alignment, mainly with valence band edge (VBE) of the absorber, resulting minimize the V_oc_ loss in PSCs [26]. Recently (in 2023), Michael Grätzel et al. synthesized a bifunctional organic molecule named CBz-PAI, and they used the designed CBz-PAI interlayer (CBz-PAI-IL) between the perovskite absorber/HTL interface to enhance the interface energy level alignment, facilitating the hole transport and simultaneously reducing the non-radiative recombination, lessening the voltage loss [27]. Their findings evidently demonstrated that the CBz-PAI interlayer containing PSC delivers an excellent efficiency of 24.7 % compared to the conventional device (22.3 %) [27]. More importantly, the CBz-PAI layer was deposited on the perovskite absorber layer and then heated at 100 °C for 5 min, which is a huge advantage because it also opens the door for using this CBz-PAI layer in flexible PSCs.

In addition to the effect of the CBz-PAI interlayer on PSC performance, further improvements and more comprehensive investigations, especially with various HTLs, are essential to improving PSC efficiency. Achieving a well-matched band alignment and high carrier mobility is crucial for facilitating the efficient transfer of photo-generated charges at the interface between the transport layers (ETL & HTL) and perovskite absorber [28]. In recent times, apart from the traditional HTL, such as Spiro-OMeTAD [15], a bunch of newly used HTLs in PSCs, namely Se/Te-doped Cu_2_O (Se/Te: Cu_2_O**)** [29], copper gallium oxide (CuGaO_2_) [30], Vanadium pentoxide (V_2_O_5_) [31], copper(I) thiocyanate (CuSCN) [32], are demonstrated an excellent PCE. For example, Liang Luo et al. results showed that Se/Te: Cu_2_O-HTL PSC offers higher efficiency (22.5 %) than the Spiro-OMeTAD-HTL device (18.5 %) [29]. Amal Bouich et al. investigations confirm that the conventional PSC efficiency (i.e., with Spiro-OMeTAD-HTL, 19.5 %) is lesser compared to the CuGaO_2_**-**HTL device (22.9 %) [30]. The findings of Saeid Asgharizadeh et al. demonstrated that the CuSCN-HTL PSC achieved an efficiency of 22.7 % more than the conventional HTL device [33]. Multiple recent reports justified that the V_2_O_5_ is employed as an HTL and interlayer between the perovskite absorber/HTL interface to attain high solar cell performance [[34], [35], [36], [37]].

The drift-diffusion SCAPS-1D simulation software has recently been widely used to investigate the impact of each layer's parameters (thickness, bandgap, defect density, e^−^ and h^+^ mobilities, etc.) on the PSC performance. Besides spending a lot of our time and money before advancing in actual device fabrication, drift-diffusion SCAPS-1D simulation results are highly reliable and consistent with the experimental work [15]. As far as we know, very few SCAPS-1D simulation reports (with/without experimental findings) are available in flexible PSC (FPSC) [[38], [39], [40], [41], [42]]. For example, A A Goje et al. (in 2023) investigated the FPSC performance with n-i-p device architecture (i.e., PET/ITO/PCBM/MABiI_3_/Spiro-OMeTAD/Ag) and demonstrated an efficiency of 18.8 %, respectively [39]. More interestingly, Chandani Dubey et al. used a ZnO nanorods-ETL (ZnO NRs) with PET/FTO/AZO/ZnO-NRs/Perovskite/PTAA/Au configuration, and they achieved a higher FPSC performance of 26.6 % [42]. Also, Feriel Bouhjar et al. employed a cobalt-doped ZnO NR-ETL in FPSC (i.e., PET/ITO/Co–ZnO/MAPbI_3_/Spiro-MeOTAD/Au) to attain the PCE of 7 % [41]. Both results [41,42] explain that the ZnO nanostructured ETL facilitates charge carrier transport (i.e., e^−^) at the ETL/perovskite absorber interface, which is highly beneficial for achieving a higher PCE in FPSC.

Therefore, in this work, initially, we choose to simulate the flexible PSC with the following architecture, PET/ITO/AZO/ZnO nanowires (ZnO NWs)/FACsPbBrI_3_/Spiro-OMeTAD/Au (as a reference device) using the version 3.3.10 drift-diffusion SCAPS-1D software. Mainly, we introduced the CBz-PAI interlayer in between the perovskite/HTL interface (i.e., PET/ITO/AZO/ZnO NWs/FACsPbBrI_3_/CBz-PAI/Spiro-OMeTAD/Au) to investigate the impact and to understand the device physics. On top of that, we have tested four other different HTLs, such as Se/Te: Cu_2_O, CuGaO_2_, V_2_O_5_ and CuSCN, in the same device configuration, especially with and without CBz-PAI interlayer, to understand the impact on the FPSC performance and to govern the most effective combination. Furthermore, we investigated the influence of perovskite absorber thickness and defect density as well as the impact of series (R_s_) and shunt (R_shunt_) resistances and working temperature on the FPSC efficiency.

## 2 Computational details

**Fig. 1:**
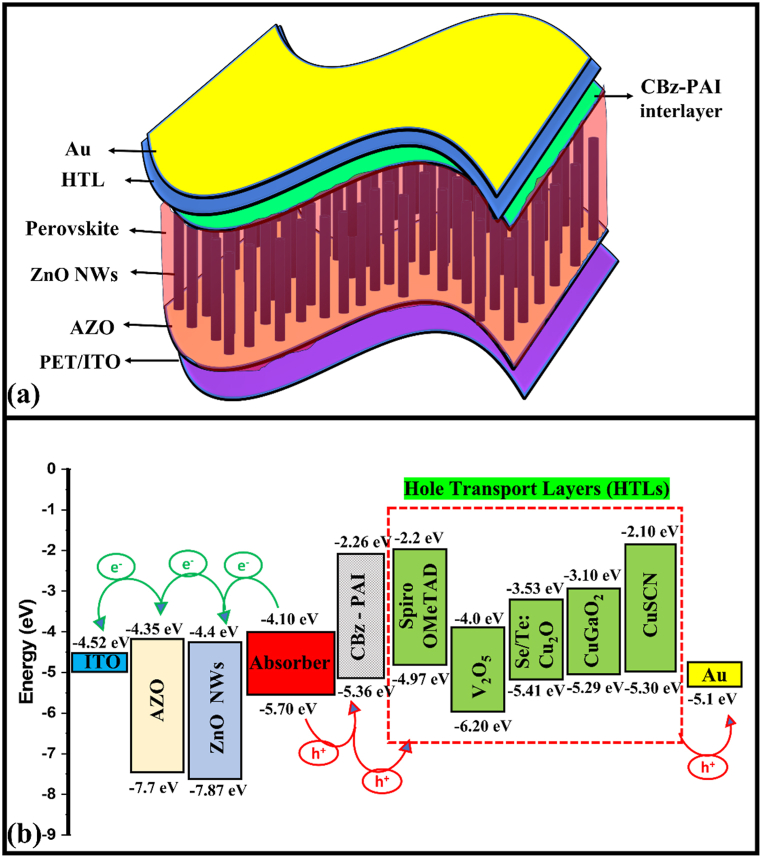
(a) The proposed FPSC structure (the inset shows CBz-PAI structure). (b) Band alignments for all the layers (ETL, absorber, and HTLs).

The freely available drift-diffusion SCAPS-1D simulation package was established by Marc Burgelman et al. [43], and it was initially designed for CdTe and CIGS solar cells. Recent improvements make the program useful to several other solar cells, such as GaAs, silicon, and perovskite solar cells. This software mainly runs and demonstrates the solar cell results, such as current density-voltage (J-V), capacitance-voltage (C–V), and quantum efficiency (QE) plots based on Poisson's, the carrier continuity, and the drift-diffusion equations [43]. In this study, we initially constructed a reference FPSC with an n-i-p configuration containing the conventional Spiro-OMeTAD-HTL (without CBz-PAI interlayer), and later, the same device simulated with CBz-PAI interlayer, as shown in Fig. 1a. The proposed FPSC consists of PET/ITO, AZO, ZnO NWs, FACsPbBrI_3_, Spiro-OMeTAD (with/without CBz-PAI interlayer) and Au as the TCO, seed layer, NWs-based-ETL, absorber, HTL and contact. Furthermore, four different HTLs (i.e., Se/Te: Cu_2_O, CuGaO_2_, V_2_O_5_ and CuSCN) were systematically tested in the same device configuration, especially with and without the CBz-PAI interlayer. The corresponding energy levels diagram of the constructed FPSC is shown in Fig. 1b. The simulation physical input parameters, including the values of interface defects for all the proposed layers, are displayed in Table 1, Table 2, Table 3, and the physical input parameters were gathered from reported experimental and computational papers [15,[28], [29], [30], [31], [32],^39^,^41^,^42^,^44^,^45^]. All the simulations were done at 300 K under one sun irradiation with integrated power density 1000 W m^−2^ (AM1.5G) according to standard solar cell test conditions [46].

**Table 1:** Physical input parameters of reference FPSC.

Parameters	ITO (TCO)	AZO (ETL)	ZnO NW (ETL)	Perovskite (Absorber)	CBz-PAI (IL)
**Thickness (nm)**	500	100	800	500	5
**E**_**g**_**(eV)**	3.65	3.50	3.22	1.59	3.1
**χ (eV)**	4.80	4.65	4.50	4.09	2.26
$\varepsilon_{r}$	8.90	9.00	9.00	6.6	3.0
$N_{c}$**(cm**^**−**^**^3^)**	5.2 × 10^18^	2.2 × 10^18^	2.2 × 10^18^	2.0 × 10^19^	2.2 × 10^18^
$N_{v}$**(cm**^**−**^**^3^)**	1.0 × 10^18^	1.8 × 10^19^	1.8 × 10^19^	2.0 × 10^18^	1.8 × 10^19^
$\mu_{n}$**(cm**^**2**^**V**^**−**^**^1^s**^**−**^**^1^)**	1E12	100	100	8.106	2.0 × 10^−2^
$\mu_{p}$**(cm**^**2**^**V**^**−**^**^1^s**^**−**^**^1^)**	1E12	25	25	1.8	2.0 × 10^−2^
$N_{D}$**(cm**^**−**^**^3^)**	1.0 × 10^12^	1.0 × 10^15^	1.0 × 10^18^	1.3 × 10^16^	0
$N_{A}$**(cm**^**−**^**^3^)**	0	1	1	1.3 × 10^16^	1.0 × 10^19^
$N_{t}$**(cm**^**−**^**^3^)**	1.0 × 10^14^	1.0 × 10^16^	1.0 × 10^16^	4 × 10^13^	0
**Reference**	[39]	[42]	[41,42,44,45]	[15]	[28]

**Table 2:** SCAPS-1D physical input parameters of various HTLs for FPSC.

Parameters	Spiro-OMeTAD	Se/Te: Cu_2_O	CuGaO_2_	V_2_O_5_	CuSCN
**Thickness (nm)**	150	100	100	100	100
**E**_**g**_**(eV)**	2.9	1.88	2.51	2.4	3.4
**χ (eV)**	2.2	3.53	3.10	3.1	2.1
$\varepsilon_{r}$	3.0	7.11	3.0	8	10
$N_{c}$**(cm**^**−**^**^3^)**	2.2 × 10^18^	2.02 × 10^17^	1.0 × 10^19^	2.2 × 10^18^	2.5 × 10^18^
$N_{v}$**(cm**^**−**^**^3^)**	2.2 × 10^18^	1.17 × 10^19^	1.0 × 10^19^	1 × 10^19^	1.8 × 10^19^
$\mu_{n}$**(cm**^**2**^**V**^**−**^**^1^s**^**−**^**^1^)**	1.0 × 10^−4^	1297	0.2	150	2 × 10^−4^
$\mu_{p}$**(cm**^**2**^**V**^**−**^**^1^s**^**−**^**^1^)**	1.0 × 10^−4^	1297	0.2	100	2 × 10^−4^
$N_{D}$**(cm**^**−**^**^3^)**	0	0	0	0	0
$N_{A}$**(cm**^**−**^**^3^)**	1.0 × 10^18^	3.0 × 10^18^	1.0 × 10^18^	10^19^	1.0 × 10^17^
$N_{t}$**(cm**^**−**^**^3^)**	1.0 × 10^15^	1.0 × 10^15^	1.0 × 10^15^	1.0 × 10^15^	1.0 × 10^8^
**Reference**	[15]	[29]	[30]	[31]	[32]

**Table 3:** SCAPS-1D physical input parameters for interfaces.

Interface	Defect type	Capture Cross Section: e^−^/h^+^ (cm^2^)	Energetic Distribution	Reference for defect energy level	Total density (cm^−2^) (integrated over all energies)
ETL/FACsPbBrI_3_	Neutral	1.0 × 10^−19^ 1.0 × 10^−19^	Single	Above the VB maximum	1.0 × 10^10^
FACsPbBrI_3_/CBz-PAI	Neutral	1.0 × 10^−19^ 1.0 × 10^−19^	Single	Above the VB maximum	1.0 × 10^10^
FACsPbBrI_3_/HTL	Neutral	1.0 × 10^−18^ 1.0 × 10^−19^	Single	Above the VB maximum	1.0 × 10^10^

## 3 Results and discussions

### 3.1 Effect of CBz-PAI interlayer on FPSCs performance

**Fig. 2:**
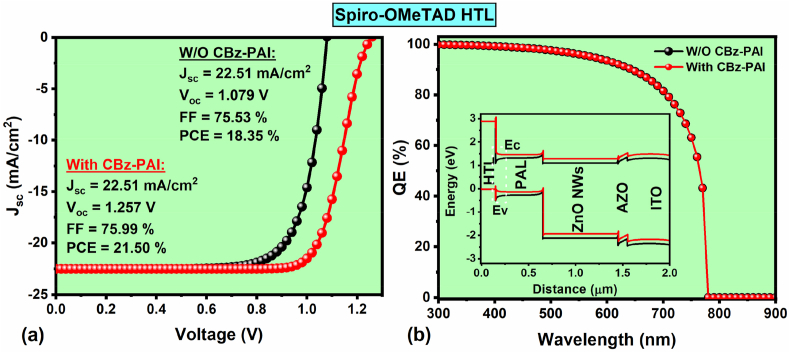
(a) J–V characteristics (b) QE plots (inset: energy band diagram) of the simulated reference FPSC using conventional Spiro-OMeTAD-HTL with and without CBz-PAI interlayer.

Initially, we designed and simulated the reference FPSC using n-i-p architecture (i.e., PET/ITO/AZO/ZnO NWs/FACsPbBrI_3_/Spiro-OMeTAD/Au) without CBz-PAI interlayer, and the corresponding device delivered an efficiency of 18.35 % (Fig. 2a). The resulting J-V characteristics (with respective J_sc_, V_oc_, FF & PCE) and QE plot, including the band diagram (figure inset) of the reference FPSC, are displayed in Fig. 2a and **b**. Later, the CBz-PAI interlayer was added to the perovskite/HTL interface to investigate the influence on FPSC performance. Interestingly, after incorporating the CBz-PAI interlayer into the classical Spiro-OMeTAD-HTL-based FPSC, the modified device demonstrated a highly enhanced PCE from 18.35 % to 21.5 %, as shown in Fig. 2a. Mainly, the device V_oc_ expanded from 1.08 to 1.26 V due to the enhanced perovskite/HTL interface. The corresponding device QE plots and the energy band diagrams with and without CBz-PAI interlayer are shown in Fig. 2b and insets. In Fig. 1b, it is evident that the conventional FPSC (i.e., with Spiro-OMeTAD-HTL) demonstrates a significant VBE/HOMO energy level misalignment of 730 meV. In contrast, the CBz-PAI-IL FPSC shows a HOMO level of 340 meV, which is above the absorber VBE, due to that it facilitates (blocks) the hole (electron) injection from the absorber before they reach the HTL. Because of the reduced VBE/HOMO level mismatch (i.e., 340 meV), the primary interfacial e^−^-h^+^ recombination occurs at the perovskite absorber/CBz-PAI-IL interface, which indicates that the V_oc_ losses at this interface are smaller than at the perovskite absorber/Spiro-OMeTAD-HTL interface [28]. According to Michael Grätzel et al. discussion, due to the large VBE misalignment, a robust h^+^ accumulation within the HTL and e^−^ gathering in the absorber layer at the perovskite absorber/HTL interface is generated, which directs to a substantial increment in non-radiative recombination at the perovskite absorber/HTL interface [28]. This mentioned behavior possibly occurred in the reference FPSC (without CBz-PAI-IL) compared to the CBz-PAI interlayer consisted device, and it is evident that the addition of the CBz-PAI interlayer reduces the recombinations and significantly increases the FPSC performance. The extracted total recombination rate response at the perovskite/HTL interface for both FPSCs (with and without interlayer) is shown in Fig. S1a, which also confirms that the addition of CBz-PAI interlayer significantly modifies the recombination bahavior. Therefore, we choose four other HTLs, such as Se/Te: Cu_2_O, CuGaO_2_, V_2_O_5_ and CuSCN, to test their impact on the proposed FPSC PV performance, especially with and without CBz-PAI interlayer to find the suitable device design.

### 3.2 Effect of CBz-PAI interlayer on various HTLs-based FPSCs performance

**Fig. 3:**
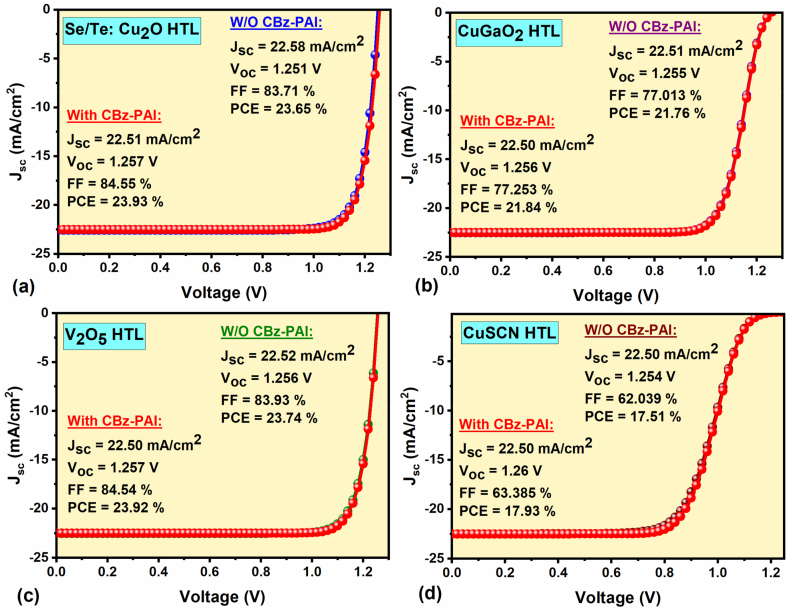
J–V characteristics of (a) Se/Te: Cu_2_O-HTL, (b) CuGaO_2_-HTL, (c) V_2_O_5_-HTL, and (d) CuSCN-HTL FPSCs with and without CBz-PAI interlayer.

In this sub-section, the FPSC performance was determined with various HTLs (i.e., Se/Te: Cu_2_O, CuGaO_2_, V_2_O_5_ and CuSCN) instead of conventional Spiro-OMeTAD-HTL with and without CBz-PAI interlayer. The thickness of all chosen HTLs is 100 nm, as specified in Table 2, and all the parameters of other respective layers of the simulation remain the same as the reference device. The obtained results show that all the chosen HTL-based FPSCs without CBz-PAI interlayer demonstrate an efficiency of 23.65 % (Se/Te: Cu_2_O-HTL), 21.76 % (CuGaO_2_-HTL), 23.74 % (V_2_O_5_-HTL), and 17.51 % (CuSCN-HTL), respectively. The corresponding FPSCs J-V characteristics with their respective PV parameters (J_sc_, V_oc_, FF & PCE) are displayed in Fig. 3a–d. After adding the CBz-PAI interlayer between the perovskite/HTL interface, the FPSCs performances were significantly enhanced, for example, from 23.65 % to 23.93 % for Se/Te: Cu_2_O-HTL, from 21.76 % to 21.84 % for CuGaO_2_-HTL, from 23.74 % to 23.92 % for V_2_O_5_-HTL and from 17.51 % to 17.93 % for CuSCN-HTL device, as shown in Fig. 3a–d. Interestingly, due to the interface between the perovskite and HTL, as well as the ohmic contact between the HTL and the metal electrode, the FF values are changed, for example, 84.5 % for Se/Te–Cu_2_O and V_2_O_5_, 77 % for CuGaO_2_ and 63 % for CuSCN HTL-based devices; however, all devices with and without CBz-PAI interlayer showed almost similar J_sc_ and V_oc_ behavior. Moreover, the FPSC performance enhancement does not solely rely on the VBE of HTL but also on several factors, such as the hole mobility of HTL (in our case) and the ohmic contact between HTL and the metal electrode, surface/interface issues (in actual device), etc. As a result, Se/Te: Cu_2_O and V_2_O_5_ HTLs-based FPSCs offer a high and excellent PCE of nearly 24 % with the CBz-PAI interlayer compared to the other three FPSCs, including reference device (i.e., CuGaO_2_, CuSCN and Spiro-OMeTAD HTL devices).

**Fig. 4:**
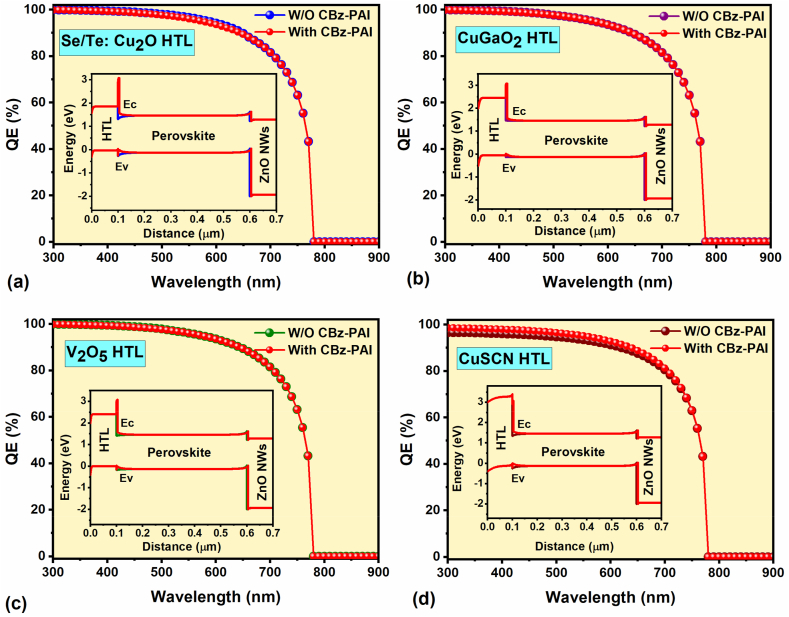
QE plots (insets: energy band diagram) of (a) Se/Te: Cu_2_O-HTL, (b) CuGaO_2_-HTL, (c) V_2_O_5_-HTL and (d) CuSCN-HTL FPSCs with and without CBz-PAI interlayer.

The corresponding FPSCs QE plots and the energy band diagrams (insets) with and without CBz-PAI interlayer are shown in Fig. 4a–d. Noticeably, the energy level alignment is identical on the ETL/absorber side for all the proposed HTL-based FPSCs, whereas the main difference is observed on the perovskite/HTL side. Fig. 1b confirms that (without CBz-PAI interlayer), the V_2_O_5_ (500 meV), CuGaO_2,_ and CuSCN HTLs (almost 400 meV) demonstrate a significant VBE/HOMO energy level misalignments than Se/Te: Cu_2_O HTL (below 300 meV). Besides the energy level alignment, the chosen HTL properties, such as hole mobility and hole-extracting ability, smooth the hole transport from the perovskite absorber. As we discussed before, after adding the CBz-PAI interlayer, the charge carrier recombinations are highly reduced at the absorber/HTL interface, diminishing the V_oc_ loss in all the proposed HTL-based FPSCs (insets: Fig. 3a–d). Figs. S1b–e clearly demonstrates that the total recombination rate at the interface is considerably reduced for all HTLs with CBz-PAI interlayer compared to the normal ones (i.e., without interlayer), resulting in enhanced PCE. Therefore, based on the obtained PV parameters, especially after testing all chosen HTLs-based FPSCs with and without CBz-PAI interlayer, the Se/Te: Cu_2_O-HTL-based FPSC delivered a higher performance. As a result, Se/Te: Cu_2_O-HTL is chosen for further FPSC optimization.

### 3.3 Impact of absorber thickness and defect density on FPSCs performance

**Fig. 5:**
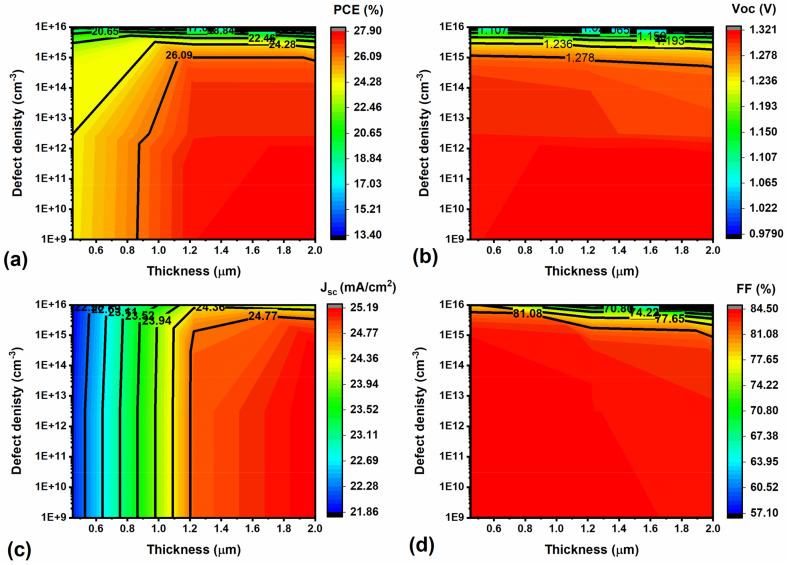
Impact of perovskite absorber thickness and N_t_ on the FPSC performance.

In general, the absorber thickness plays a vital role in PSCs, which mainly impacts photon collection and solar cell performance. In addition, in actual solar cells, Schottky, Frenkel and intrinsic point defects (i.e., vacancy & interstitial defects) are usually presented at the surface or interface or grain boundaries, significantly affecting the perovskite absorber material electrical properties [47]. Therefore, to understand the effect of chosen absorber thickness and defect density (N_t_) on FPSC performance, the thickness and N_t_ were simultaneously adjusted from 0.45 μm to 2 μm and from 1 × 10^9^ cm^−3^ to 1 × 10^16^ cm^−3^, respectively. The corresponding PV parameter results (J_sc_, V_oc_, FF & PCE) are displayed using contour maps in Fig. 5a–d. It is shown that when the N_t_ was smaller than 1 × 10^15^ cm^−3^ and at the same time the perovskite layer thickness was more than 1.5 μm, the corresponding FPSC offers the highest PCE of 27.90 %, which confirms that increased thickness enhances photon absorption (i.e., generation of more electron-hole pairs) and with reduced defects (lower N_t_), resulting in improved FPSC efficiency (Fig. 5a). The higher-performance FPSC demonstrated the V_oc_ of 1.32 V (i.e, N_t_ = lower than 1 × 10^12^ cm^−3^), and while increasing the N_t_ (i.e, N_t_ = 1 × 10^16^ cm^−3^), the V_oc_ is significantly reduced by 1.32 V–0.98 V, impacting and diminishing the device PCE to almost 14 %, due to enhanced defects in the device. Moreover, the FF decreases gradually from 84.50 % to 57.10 % for the higher N_t_, as shown in Fig. 5d. According to previous reports [48,49], an adjustment in the N_t_ usually modifies the defects, and mainly for higher N_t_, it reduces the τ_n,p_ (i.e., charge carriers lifetime), and L_n_ and L_p_ (i.e., diffusion lengths), which are highly responsible for surface/interface recombinations, reducing the FPSC efficiency. Hence, it is evident that the lesser N_t_ (i.e., reduced defects) and appropriate absorber thickness (to achieve higher photons) are the keys to attaining the maximum device efficiency.

Furthermore, the impact of perovskite/CBz-PAI interface defect density on the FPSC performance is investigated. For this study, the N_t_ is varied from 1 × 10^10^ cm^−3^ to 1 × 10^20^ cm^−3^, and the corresponding PV parameters are displayed in Table S1. It is evident that the increment in the perovskite/CBz-PAI interface defect density significantly impacts the FPSC V_oc_, for example, 1.32 V for 1 × 10^10^ cm^−3^ and it reduced to 1.11 V for 1 × 10^20^ cm^−3^, resulting in a considerable decrement in the efficiency from 27.87 % to 24.35 %, respectively. As a result, the lower Nt of 1 × 10^10^ cm^−3^ is opted for further studies. In addition, the interlayer thickness is altered from 5 nm to 20 nm to understand the impact, and surprisingly, the FPSC performance has not considerably changed. However, it will influence the real FPSC due to surface/interface issues similar to Z. Hawash et al. report [50].

### 3.4 Effect of parasitic resistances (R_S_ and R_sh_) working temperature on FPSC performance

**Fig. 6:**
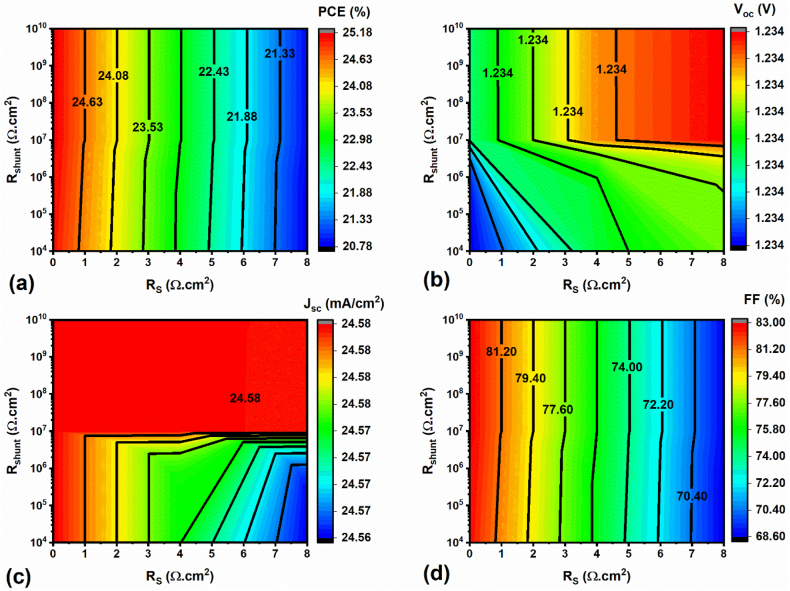
PV parameters of the FPSC using various R_S_ and R_sh_.

Usually, R_S_ and R_sh_ resistances, also called parasitic resistances, play a crucial part in PSCs, and the relevant R_S_ and R_sh_ results obtained from J-V curves usually assist in understanding the losses within the device. The highest PCE experimental device generally demonstrates lower R_S_ (i.e., mainly hinge on the contacts between ETL & HTL as well as metal contacts) and higher R_sh_ (i.e., depends on the manufacturing defects, mainly surface/interface defects, such as pinholes/voids, etc.) values [15]. Hence, the influence of R_S_ and R_sh_ was thoroughly explored by varying R_s_ from 0 to 8 Ω cm^2^ and R_sh_ from 10^3^ to 10^10^ Ω cm^2^. The respective PV parameters (J_sc_, V_oc_, FF & PCE) are shown using contour maps in Fig. 6a–d. After including R_S_ & R_sh_ into the non-ideal FPSC, the obtained results demonstrate that the FPSC efficiency significantly reduces from 27 % to 21 % (Rs = 8 Ω cm^2^, Fig. 6a), whereas the FF also diminishes from 84 % to 69 % (Fig. 6d), similar to our previous reports [15,47,51,52]. Noticeably, V_oc_ (1.32–1.24 V) and J_sc_ (25.2–24.6 mA/cm^2^) reduced a little while adjusting R_S,_ whereas they remained unchanged while varying both R_S_ and R_sh_. Therefore, it is evident that there are more chances for enhancing recombinations with high R_s_ and low R_sh_, leading to lower device efficiency. For example, Michael Grätzel et al. investigated and showed the impact of R_s_ based on two different interlayers at the perovskite absorber/HTL interface, such as PEAI & CBz-PAI [28]. Their results evidently display that the CBz-PAI layer has a HOMO level (i.e., 40 meV) above the perovskite absorber VBE (i.e., excellent h^+^ transfer by the CBz-PAI-IL into the HTL). On the contrary, the PEAI layer has a HOMO level (i.e., 50 meV) which is below the perovskite absorber VBE and forms a barrier, in consequence, in an enormous Rs in the device [28].

**Fig. 7:**
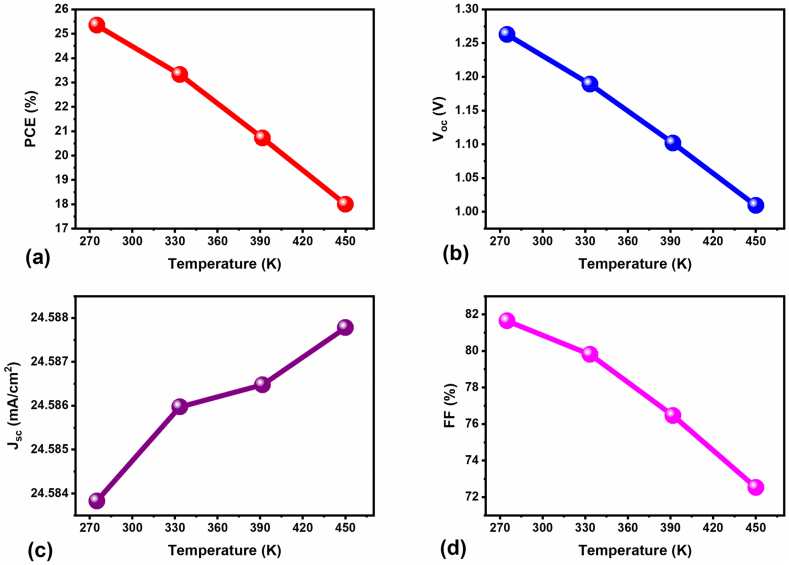
Change of FPSC PV parameters as a function of various operating temperatures.

Working temperature is a key environmental factor that effectively influences FPSC performance [53]. Therefore, we adjusted the working temperature range from 270 to 450 K to investigate its impact on FPSC performance (we used a non-ideal device, i.e., with R_S_ and R_sh_), and the corresponding device results are illustrated in Fig. 7a–d. It is clear that increasing the operating temperature affects all the FPSC PV parameters, mainly the FF & V_oc_, as shown in Fig. 7a–d. The FF and V_oc_ values are drastically reduced from 82 % to 72 % and 1.26 V–1 V, whereas J_sc_ increases marginally with temperature (from 22.583 to 22.587 mA/cm^2^), respectively. As we discussed in our previous report [51], the higher operating temperatures specifically impact the V_oc_; moreover, it possibly induces the e^−^'s thermal excitation, triggering some juddering and subsequent instability, resulting in charge carrier recombinations [54].

**Fig. 8:**
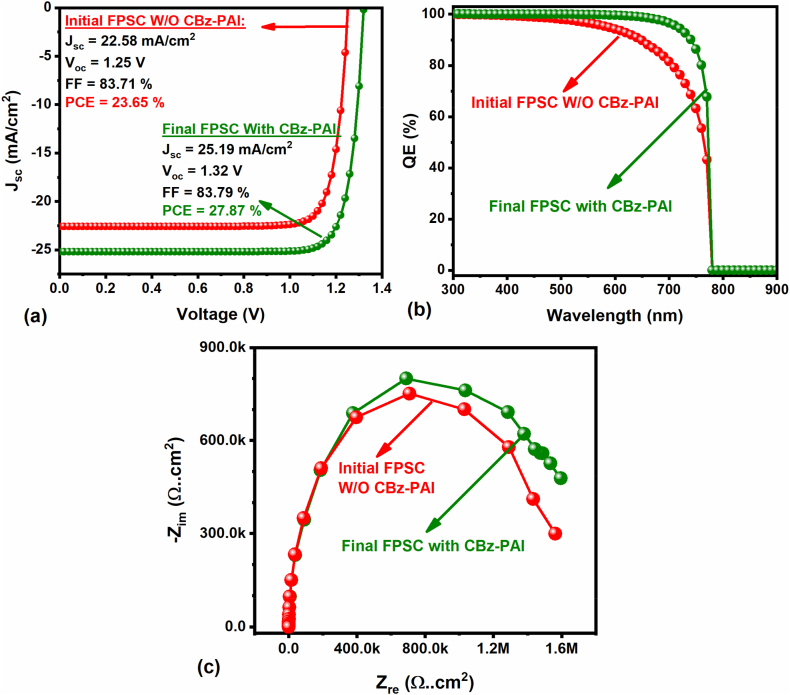
(a) J–V curves (inset: PV parameters), (b) QE and (c) Nyquist plots of the Se/Te: Cu_2_O-HTL-based initial (without CBz-PAI interlayer) and final (with CBz-PAI interlayer) FPSCs.

The J-V curves as well as QE plots of the preliminary (without CBz-PAI interlayer) and optimized (with CBz-PAI interlayer) Se/Te: Cu_2_O-HTL-based FPSCs are demonstrated in Fig. 8a and **b**. It is clear that, after an organised optimization, the FPSC with CBz-PAI interlayer showed highly enhanced PCE from 23.6 % to 27.8 %. Likewise, the final optimized FPSC QE image exhibits an enhancement of nearly 100 % in the wavelength range of 300 to almost 700 nm than the initial FPSC (without a CBz-PAI interlayer). In addition, the extracted Nyquist plot is displayed in Fig. 8c for the initial and final FPSCs. It demonstrates that the significant expansion observed in the semicircle diameter for the final FPSC than the initial device indicates an efficient hole transport and reduced recombination at the perovskite/HTL interface (with interlayer), similar to our previous report [47,52,55]. Hence, both (initial & final) FPSCs PCE, as well as QE images, confirm the significance of the CBz-PAI interlayer at the perovskite/HTL interface, including appropriate optimized parameters (i.e., thickness and defect density), which are essential to achieve maximum efficiency.

Table 4 demonstrates the PV performance comparison of published experimental as well as theoretical reports on FPSC with this proposed work, explicitly using ZnO NWs/NRs-ETL. Interestingly, the PET/ITO/AZO/ZnO NWs/FACsPbBrI_3_/CBz-PAI/Se/Te: Cu_2_O/Au device architecture mainly with CBz-PAI interlayer at perovskite/HTL interface offers the highest PCE of nearly 28 % due to the enhanced hole transport, which is higher than other available reports (see Table 4), especially with Chandani Dubey et al. report (26.63 %) [42]. It is worth mentioning here that in their simulated FPSC, they used a lanthanide-doped perovskite absorber (i.e., Ln^3+^ - MAPbI_3_) with a bandgap value of 1.3 eV, which absorbs more photons than this proposed work (i.e., here the absorber bandgap is 1.59 eV). Even though with this wider bandgap, the designed FPSC delivered an excellent efficiency (i.e., 28 %), and this confirms the possibility of enhancing the FPSC efficiency further (i.e., over 30 %) in the near future using the same proposed device design with various potential perovskite absorbers, including suitable ETL and HTLs, especially with CBz-PAI interlayer. Noticeably, this proposed work comprehensively focused on the perovskite/HTL interface enhancement using CBz-PAI interlayer; however, according to the published reports, mainly using ZnO NWs ETL-based real FPSCs still not exceeding the PCE of 15 % [56] due to several reasons, such as higher recombinations formed at the ZnO NWs/perovskite interface or the contact between ZnO NWs-ETL into the HTL (i.e., due to larger NWs length or lower perovskite absorber thickness) [57,58], etc. Therefore, further insightful studies are needed, mainly on the ZnO NWs/perovskite interface using passivation/interlayer similar to this study to enhance the FPSC efficiency further. For example, Muhammad Fahim et al. used the ZnS-IL between ZnO NRs-ETL/perovskite absorber interface to improve the energy band alignment and to facilitate the electron transfer, resulting in an improved PCE of 14.7 % than conventional FPSC (12 %, without ZnS interlayer) [56].

**Table 4:** Comparison of reported experimental and theoretical FPSCs efficiency.

Device	J_sc_ (mA/cm^2^)	V_oc_ (V)	FF (%)	PCE (%)	Ref
Substrate-1/ZnO/ZnO NR/Perovskite/Spiro-OMeTAD/Au	07.52	0.80	43.0	02.60	Exp [59]
Substrate-1/ZnAc/ZnO NW/Perovskite/Spiro-OMeTAD/Au	14.42	0.68	65.0	06.40	Exp [60]
Substrate-2/AZO/ZnO-NRs/MASnI_3_/PTAA/Au	22.03	1.38	87.5	26.63	Sim [42]
Substrate-1/ZnO/ZnO NW/Perovskite/Spiro-OMeTAD/Au	23.50	0.95	59.0	12.80	Exp [61]
Substrate-1/Co:ZnO NR/Perovskite/Spiro-OMeTAD/Au	14.30	1.04	47.0	07.00	Exp [41]
Substrate-1/ZnO/ZnO NR/CsFAMAPb(IBr)_3_/Spiro-OMeTAD/Ag	24.10	0.91	63.0	13.86	Exp [56]
Substrate-1/ZnO/core-shell ZnO@ZnS NRs/CsFAMAPb(IBr)_3_/Spiro-OMeTAD/Ag	24.40	0.97	63.0	14.68	
Substrate-1/AZO/ZnO NWs/FACsPbBrI_3_/CBz-PAI/Se/Te: Cu_2_O/Au	25.19	1.32	83.79	27.86^a^	This work

a Substrate-1 = PET/ITO, Substrate-2 = PET/FTO, Perovskite = MAPbI_3_, Exp = Experimental, Sim = Simulation.

## 4 Conclusion

In this study, we systematically examined the performance of flexible PSCs, specifically, the impact of the CBz-PAI interlayer at the perovskite absorber/HTL interface using a drift-diffusion SCAPS-1D simulation. Initially, we tested the FPSC performance by adopting conventional Spiro-OMeTAD HTL with and without the CBz-PAI interlayer, and it confirms that the interlayer device delivers an enhanced PCE compared to the normal one. The CBz-PAI interlayer shows a suitable and reduced HOMO level (340 meV) over the perovskite absorber VBE than a conventional device (730 meV), which facilitates (blocks) the hole (electron) injection from the absorber before they reach the HTL. Additionally, it diminishes the interfacial charge carrier recombinations at the perovskite/HTL interface, resulting in reduced V_oc_ losses. Later, substantial work was carried out on different HTLs, such as Se/Te: Cu_2_O, CuGaO_2_, V_2_O_5_ and CuSCN, in the same device configuration (with and without CBz-PAI interlayer), to find suitable architecture to attain the maximum PCE. Interestingly, after optimization, the following FPSC design with CBz-PAI interlayer (PET/ITO/AZO/ZnO NWs/FACsPbBrI_3_/CBz-PAI/Se/Te: Cu_2_O/Au) demonstrates a maximum efficiency of 27.90 %, which is highest among the available FPSC reports. As a result, this proposed work provides a roadmap for developing FPSCs with improved PCE using interlayer strategy, and further insightful experimental studies (either using interlayer at ETL/perovskite absorber interface and/or perovskite absorber/HTL interface) are essential to finding the problems at the interfaces, will be beneficial to improve the FPSC efficiency.

## CRediT authorship contribution statement

**Selma Rabhi:** Writing – original draft, Visualization, Validation, Software, Investigation, Formal analysis, Data curation. **Talaat A. Hameed:** Writing – review & editing, Investigation, Formal analysis. **Sasikumar Mayarambakam:** Investigation, Formal analysis. **M. Khalid Hossain:** Investigation, Formal analysis, Data curation. **Karthick Sekar:** Writing – review & editing, Validation, Supervision, Investigation, Formal analysis, Conceptualization.

## Declaration of competing interest

The authors declare that they have no known competing financial interests or personal relationships that could have appeared to influence the work reported in this paper.
