# Supplementary material for: The impact of CBz-PAI interlayer in various HTL-based flexible perovskite solar cells: A drift-diffusion numerical study
Source: Heliyon. 2024 May 11;10(10):e31138. doi: 10.1016/j.heliyon.2024.e31138 (PMC11108980; doi:10.1016/j.heliyon.2024.e31138)
Supplement: Multimedia component 1 [file mmc1.docx]

**Supplementary information**

**The impact of CBz-PAI interlayer in various HTL-based flexible perovskite solar cells: A drift-diffusion numerical study**

Selma RABHI,^a,b^ Talaat A. Hameed,^c^ Sasikumar Mayarambakam,^d^ M. Khalid Hossain,^e^ Karthick SEKAR^f,*^

1. Laboratory of Semiconductors Material and Metallic Oxides, USTHB, Bab-Ezzouar,16111, Algiers, Algeria.
2. Dr. Yahia Fares University of Medea, Medea 26000, Algeria.
3. Solid-State Physics Department, Physics Research Institute, National Research Centre, 33 El Bohouth St., Dokki, Giza, 12622, Egypt.
4. Department of Materials Science and Engineering, Johns Hopkins University, Baltimore, Maryland 21210, USA.
5. Institute of Electronics, Atomic Energy Research Establishment, Bangladesh Atomic Energy Commission, Dhaka 1349, Bangladesh.
6. Aix-Marseille Université, CNRS, Institut Matériaux Microélectronique Nanosciences de Provence, Faculté de Saint Jérôme, 13397 Marseille Cedex 20, France.

**Table S1**. PV parameters for different Perovskite/CBz-PAI interface defect densities.

| Perovskite/CBz-PAI  interface defect density (cm^-3^) | J_sc_  (mA/cm^2^) | V_oc_  (V) | FF  (%) | PCE  (%) |
| --- | --- | --- | --- | --- |
| 1E10 | 25.18859862 | 1.320416 | 83.7938 | 27.8693 |
| 1E12 | 25.18859862 | 1.320416 | 83.7938 | 27.8693 |
| 1E14 | 25.18859825 | 1.299682 | 85.0209 | 27.8334 |
| 1E16 | 25.18856209 | 1.206761 | 88.1501 | 26.7946 |
| 1E18 | 25.18732252 | 1.121622 | 87.1265 | 24.6138 |
| 1E20 | 25.18666721 | 1.111891 | 86.9659 | 24.3547 |





**Figure S1.** Total recombination rate bahavior of (a) Spiro-OMeTAD-HTL, (b) Se/Te: Cu_2_O-HTL, (c) CuGaO_2_-HTL, (d) V_2_O_5_-HTL and (e) CuSCN-HTL FPSCs with and without CBz-PAI interlayer, respectively.
